# Anti-Septic Functions of Cornuside against HMGB1-Mediated Severe Inflammatory Responses

**DOI:** 10.3390/ijms23042065

**Published:** 2022-02-13

**Authors:** Nayeon Kim, Chaeyeong Kim, Soo Ho Ryu, Wonhwa Lee, Jong-Sup Bae

**Affiliations:** 1 College of Pharmacy, Research Institute of Pharmaceutical Sciences, Kyungpook National University 80 Daehak-ro, Buk-gu, Daegu 41566, Korea; cgullinny@naver.com (N.K.); rlacodud1213@naver.com (C.K.); rsh604@gmail.com (S.H.R.); 2 Department of Chemistry, Sungkyunkwan University, 2066 Seobu-ro, Jangan-gu, Suwon 16419, Korea

**Keywords:** cornuside, HMGB1, endothelium, sepsis

## Abstract

High mobility group box 1 (HMGB1) is acknowledged to have critical functions; therefore, targeting this protein may have therapeutic effects. One example is potential antiseptic activity obtained by suppressing HMGB1 secretion, leading to the recovery of vascular barrier integrity. Cornuside (CN), which is a product extracted from the fruit of *Cornus* *officinalis* Seib, is a natural bis-iridoid glycoside with the therapeutic effects of suppressing inflammation and regulating immune responses. However, the mechanism of action of CN and impact on sepsis is still unclear. We examined if CN could suppress HMGB1-induced excessive permeability and if the reduction of HMGB1 in response to LPS treatment increased the survival rate in a mouse model of sepsis. In human endothelial cells stimulated by LPS and mice with septic symptoms of cecal ligation and puncture (CLP), we examined levels of proinflammatory proteins and biomarkers as an index of tissue damage, along with decreased vascular permeability. In both LPS-treated human umbilical vein endothelial cells (HUVECs) and the CLP-treated mouse model of sepsis, we applied CN after the induction processes were over. CN suppressed excessive permeability and inhibited HMGB1 release, leading to the amelioration of vascular instability, reduced mortality, and improved histological conditions in the CLP-induced septic mouse model. Overall, we conclude that the suppressed release of HMGB1 and the increased survival rate of mice with CLP-induced sepsis caused by CN may be an effective pharmaceutical treatment for sepsis.

## 1. Introduction

Despite recent research into the treatment of septic lethality, sepsis is still a notorious infection with severe mortality and morbidity rates following uncontrollable general immune activity, especially inflammation [1]. The mortality of sepsis leads to aggravated multi-organ failure (MOF) [2,3] due to acute and lethal hypoxemia-oriented oxygen shortage [1]. Although sepsis generally disturbs the kidney and lung function [2], severe injury in the kidney gives rise to the dysfunction of various organs belonging to the cardiac, neural, respiratory, intestinal, and excretory systems as a result of systemic inflammation [3]. Currently, the pathological characteristics and mechanisms of sepsis remain obscure and unresolved. Antigen-derived molecules participate in the progression of sepsis, causing the gradual release of various inflammatory factors from macrophages and leukocytes. For example, interleukin (IL)-1β and tumor necrosis factor (TNF)-α are released in the early phase, whereas HMGB1 is secreted in the late phase [4,5]. HMGB1, an influential factor in sepsis, is secreted from injured cells and stimulated immunocytes [6]. Unlike early septic mediators, HMGB1 remains stable and at a high level in the bloodstream for 1–1.5 days in animal models of sepsis [6,7]. Given that HMGB1 is a late phase factor in lethal sepsis [6,8], substances that inhibit the secretion of HMGB1 from damaged cells and stimulated immunocytes may be promising candidates for the treatment of lethal septic diseases.

**Figure 1:**
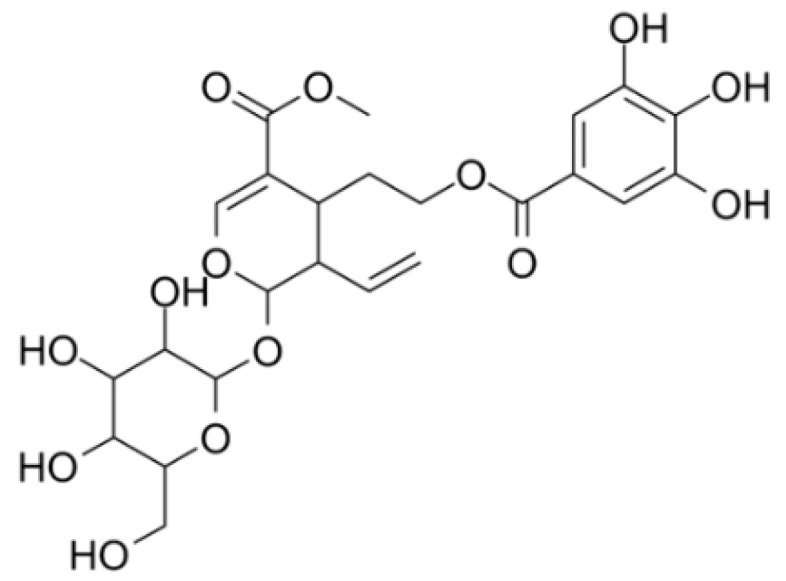
Chemical structure of cornuside (CN).

Cornuside (CN, Figure 1), a bis-iridoid glycoside, is a substance extracted from *Cornus officinalis* Sieb. et Zucc., which has been used to treat inflammatory disorder and to ameliorate hemokinesis in traditional oriental medicine. The extracts of the fruit of *C. officinalis* have a number of pharmacological effects, including antitumor, anti-inflammatory, and hepatorenal protective effects [9,10]. CN inhibits the production of proinflammatory cytokines and cell adhesion factors induced by cytokines in human endothelial cells and protects cultured rat cortical cells from oxygen-glucose deprivation [9,10]. Nevertheless, how CN interacts with sepsis and systemic inflammation mediated by HMGB1 is still unknown. Thus, we aimed to demonstrate the effectiveness of CN for preventing and treating HMGB1-mediated vascular integrity failure in vitro and in vivo.

## 2. Results and Discussion

### 2.1. CN Inhibits HMGB1 Release in LPS-Stimulated HUVECs and the CLP-Induced Sepsis Mouse Model

**Figure 2:**
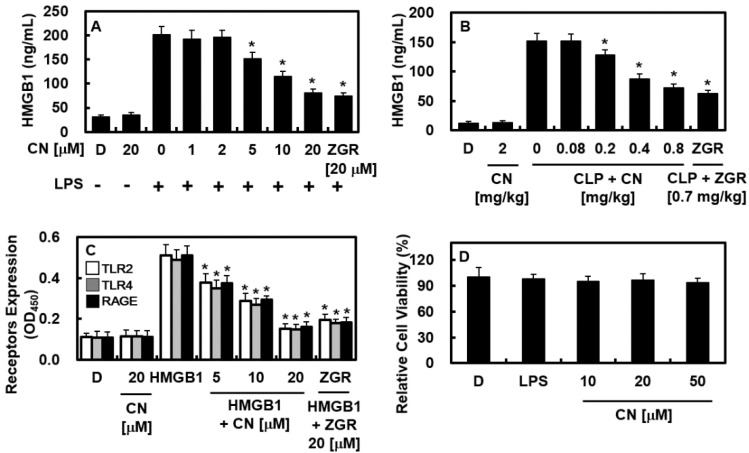
Effects of CN on release of HMGB1 and the expression levels of HMGB1 receptors. The effects of CN on HMGB1 release by: (**A**) LPS (100 ng/mL, 16 h) action in HUVECs; (**B**) CLP surgery in male mice (B, *n* = 5/each group) were determined with an ELISA; (**C**) the effects of CN on the expression levels of TLR2 (white bar), TLR4 (gray bar), and RAGE (black bar) by HMGB1 (1 μg/mL) were measured using an ELISA; (**D**) the effects of LPS (100 ng/mL) or CN on cellular viability were measured using the MTT assay. Data are shown as the mean ± SD values from three independent experiments conducted in triplicate on different days. D represents 0.2% DMSO, which was used as the vehicle control. * *p* < 0.05 versus group treated with LPS alone (**A**), CLP alone (**B**), or HMGB1 alone (**C**).

In this study, zingerone (ZGR) was uses as a positive control [11,12,13]. First, we applied LPS to activate HUVECs, and then, administered CN at doses of 1, 2, 5, 10, or 20 μM or ZGR (20 μM) for 16 h. The release of HMGB1 from cells was detected using an ELISA. The results showed that HMGB1 release was considerably increased by LPS treatment. In contrast, CN-treated cells had lower HMGB1 release after LPS treatment (Figure 2A). However, there was no further inhibitory effect above 20 μM (Supplementary Figure S1). The inhibitory activities of CN on HMGB1 release were also confirmed in CLP-induced mouse model (Figure 2B). In addition, the expression of the HMGB1 receptor proteins, such as TLR2, TLR4, and RAGE, was decreased by CN in HMGB1-treated HUVECs (Figure 2C). Finally, LPS at 100 ng/mL for 16 h or CN of various doses (10, 20, or 50 μM) for 48 h did not affect cellular viability, which was measured by MTT assay (Figure 2D). Thus, our results indicate that CN may be promising agent to modulate the early phase of sepsis through the inhibition of HMGB1 secretion.

### 2.2. Effects of CN on the Activation of SIRT1 and Acetylation of HMGB1

**Figure 3:**
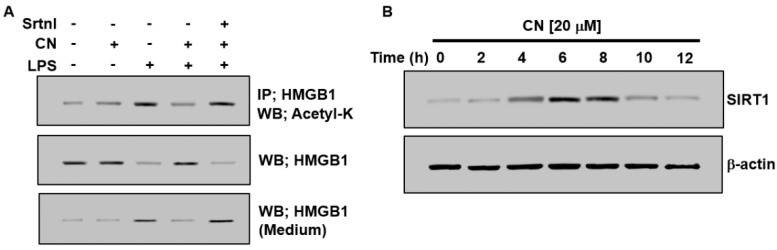
Effects of CN on the expression of SIRT1 and acetylation of HMGB1. (**A**,**B**) Cells were treated with CN (20 µM) for 0, 2, 4, 6, 8, 10, and 12 h, to study the effect of CN on the acetylation of HMGB1 and the SIRT1 expression in HUVECs. Cells were treated with LPS (100 ng/mL) with or without CN (20 µM), or with the SIRT1 inhibitor (sirtinol, Srtnl, 10 mM) for 1 h prior to CN treatment. After incubation for 6 h, the cells were lysed for immunoprecipitation. Cell lysates were subjected to immunoprecipitation and HMGB1 acetylation and the total HMGB1 protein level was measured by immunoblotting analysis using anti-acetyl-lysine (K) or anti-HMGB1 antibodies, respectively ((**A**), rows 1 and 2). After incubation for 16 h, equal volumes of medium were collected and the released HMGB1 was detected by Western blotting ((**A**), row 3). After incubation for the indicated time, the cells were lysed and analyzed via Western blotting to measure the expression levels of SIRT1 (**B**).

The binding capacity of HMGB1 to DNA or to cellular plasma depends on its excessive acetylation [14]. The hyperacetylation of serine residues in HMGB1 induces the inhibition of nuclear migration and facilitates cytoplasmic migration [15], whereas deacetylated HMGB1 is stimulated by sirtuin 1 (SIRT1); thus, HMGB1 is a novel deacetylation target for SIRT1 [16]. Therefore, we determined the effect of CN on the deacetylation of HMGB1 and the expression of SIRT1 to analyze the mechanism by which CN inhibits HMGB1 release induced by LPS. Although LPS-induced the acetylation of HMGB1, this was remarkably downregulated by treatment with CN (Figure 3A). To confirm that the role of SIRT1 was related to the suppression of HMGB1 secretion through HMGB1 deacetylation in LPS-stimulated HUVECs, we estimated the impact of the SIRT1 repressor (SIRT1 inhibitor, sirtinol [17]) on HMGB1 secretion. The results showed that stimulation with sirtinol significantly changed the effect of CN (Figure 3B) and caused excess acetylation and the subsequent release of HMGB1. Next, we investigated the impact of CN on SIRT1 expression. SIRT1 was expressed after 4 h of incubation, showed the maximum level after 6 h, continued up to 8 h, and disappeared after 12 h (Figure 3B). Overall, these results imply that CN considerably downregulated HMGB1 secretion in LPS-stimulated HUVECs via the SIRT1-related deacetylation of HMGB1.

### 2.3. CN Inhibits HMGB1-Mediated Disruption of the Vascular Barrier

**Figure 4:**
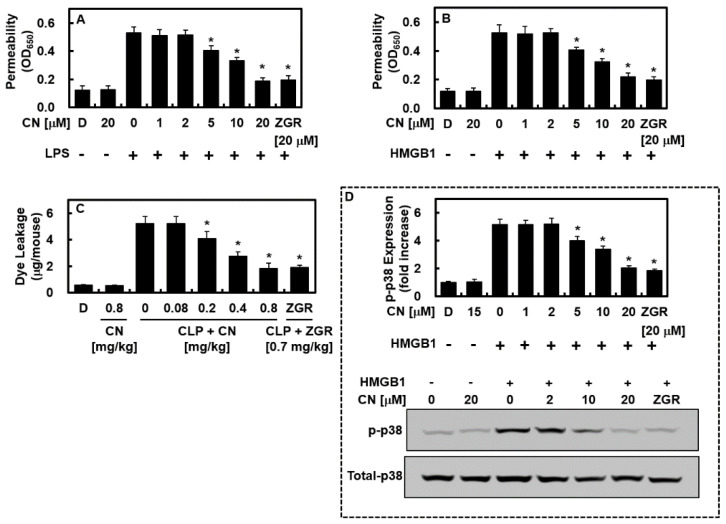
Effects of CN on HMGB1-induced permeability in vitro and in vivo. The effects of CN on: (**A**) LPS-induced hyperpermeability in HUVECs (100 ng/mL); (**B**) HMGB1-induced hyperpermeability in HUVECs (1 μg/mL); (**C**) CLP-induced hyperpermeability in mice (*n* = 5/each group); (**D**) the effects of CN on the HMGB1-mediated phosphorylation of p38 were determined using an ELISA (upper image) or Western blotting (lower image). Data are expressed as the mean ± SD values of three independent experiments on different days. * *p* < 0.05 versus group treated with LPS (**A**) or HMGB1 (**B**–**D**).

Given that a stable vascular barrier is a key to pathological vascular inflammation, and given that LPS and HMGB1 induce damage to the cohesiveness of vascular barrier [6,18,19], we performed an analysis of vascular permeability to detect the therapeutic efficacy of CN on the integrity of HUVECs. LPS-stimulated (100 ng/mL, 4 h, Figure 4A) or naïve HMGB1-treated (1 µg/mL, 16 h, Figure 4B) HUVECs were administered CN (1, 2, 5, 10, or 20 μM) or ZGR (20 μM) for 6 h. The change in barrier integrity was analyzed by observing the leakage of albumin through a single HUVEC layer that bound to Evans blue dye. CN inhibited the LPS- and HMGB1-induced excessive permeability (Figure 4A,B). The barrier-stabilizing impact of CN was determined in vivo. The mice were injected CN or ZGR intravenously 12 h after CLP induction, euthanized at 12 h after injection, and vascular leakage was investigated by detecting the quantity of Evans blue dye in abdominal rinsed fluids. CLP-derived excessive permeability was downregulated by the treatment with CN (Figure 4C). Because vascular injury induced by HMGB1 is regulated by the phosphorylation of p38 [20,21], HUVECs were stimulated by HMGB1 and treated with CN (1, 2, 5, 10, or 20 μM) or ZGR (20 μM) for 6 h, and the impact of CN or ZGR on the phosphorylation of p38 was detected using ELISA. HMGB1 increased the level of phosphorylated p38, whereas CN inhibited this effect (Figure 4D). The downregulation of HMGB1-induced barrier integrity disruption, leakage, and p38 phosphorylation indicate that CN is a promising molecule for the treatment of sepsis.

### 2.4. Effects of CN on HMGB1-Mediated Expression of CAMs, Adhesion of Neutrophils, and Migration of Leukocytes

**Figure 5:**
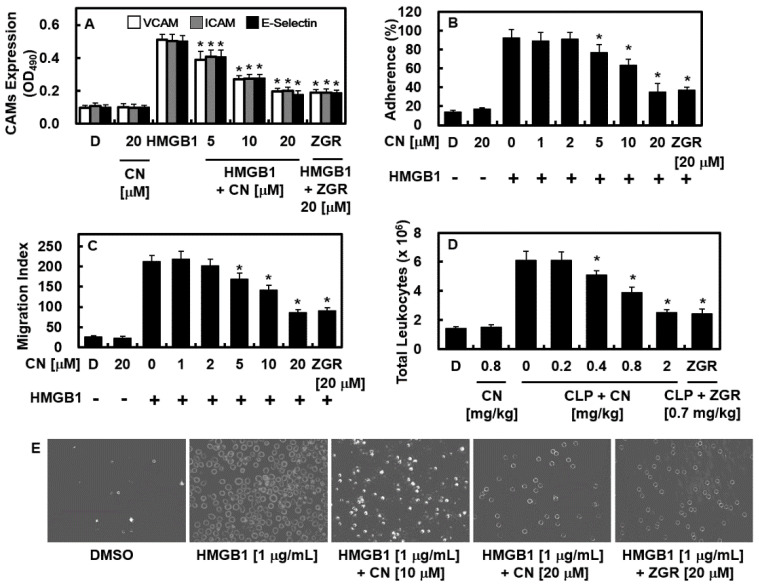
Effects of CN on HMGB1-mediated proinflammatory responses. The effects of CN on the HMGB1-induced: (**A**) expression of E-selectin (black bar), VCAM-1 (white bar), and ICAM-1 (gray bar) in HUVECs; (**B**,**E**) adherence of neutrophils to HUVEC monolayers; (**C**) migration of neutrophils through HUVEC monolayers; (**D**) the effects of CN on the CLP-induced migration of leukocytes into the peritoneal cavities of mice were analyzed by counting leukocytes in the peritoneal fluid (*n* = 5/each group); (**E**) a representative image of results presented in (**B**). Data are expressed as the mean ± SD values of three independent experiments on different days. * *p* < 0.05 versus group treated with HMGB1 or CLP.

HMGB1 induces the expression of cellular adhesive molecules, such as E-selectin, ICAM-1, and VCAM-1, on the exposed part of endothelial cells to support the translocation of immune cells to the area in which the inflammatory response occurs by passing through vascular endothelial cell layer. Therefore, the effects of CN on HMGB1-mediated expressions of CAMs, adhesion, and migration of leukocytes toward HUVECs were determined. To do this, HUVECs were incubated with HMGB1, and then treated with CN. Data showed that CN suppressed CAM expressions and adhesion and migration of leukocytes (Figure 5A–C,E). The results of the in vivo assay were identical, showing the downregulation of the HMGB1-mediated translocation of immune cells into the abdominal fluid (Figure 5D). Accordingly, these outcomes show that CN decreased the adherence and migration of inflammatory immune cells that were increased by HMGB1 stimulation.

### 2.5. CN Suppresses NF-κB/ERK Signaling and IL-1β, IL-6, and TNF-α Production

**Figure 6:**
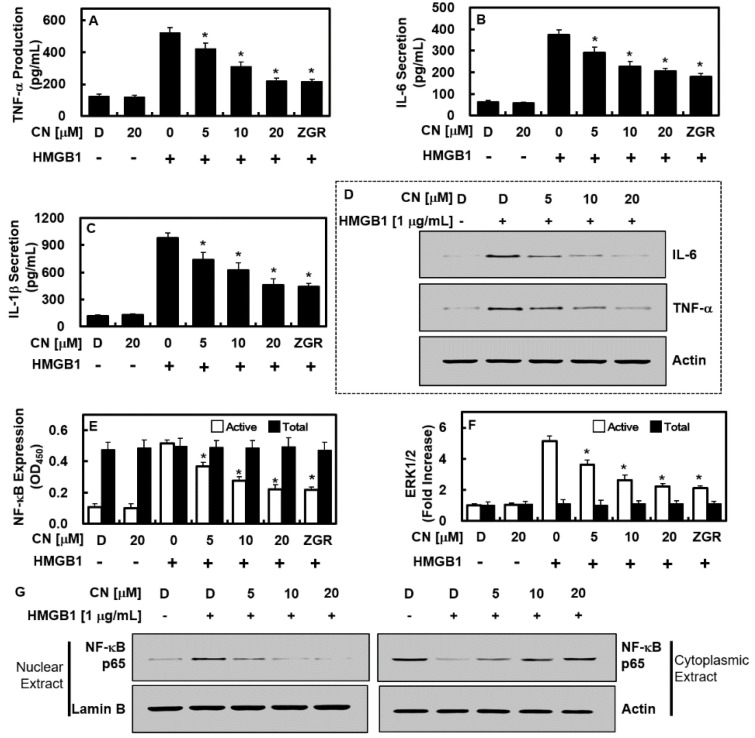
Effects of CN on the HMGB1-induced production of IL-6, TNF-α, and IL-1 and the activation of the NF-κB/ERK1/2 pathways. The effects of CN on the HMGB1-mediated production of (**A**,**D**) TNF-α, (**B**,**D**) IL-6, and (**C**) IL-1β, in the HUVECs were determined using ELISA (**A**–**C**) or Western blotting (**D**); the effects of CN on the HMGB1-mediated activation of (**E**) NF-κB p65 (white bar for phospho-p65 and black bar for total p65) and (**F**) ERK1/2 (white bar for phospho-ERK1/2 and black bar for total ERK1/2) in HUVECs; (**G**) subcellular levels of NF-κB in nuclear and cytoplasmic fractions were evaluated by Western blotting, with actin and lamin B used as loading controls for the cytoplasmic and nuclear extracts, respectively. Data are expressed as the mean ± SD values of three independent experiments on different days. * *p* < 0.05 versus group treatment Not necessary. with HMGB1 alone.

Expression of inflammatory cytokines, such as TNF-α, IL-1β, and IL-6 is induced by HMGB1. The induced inflammatory cytokines further exacerbate the situation of sepsis through the NF-κB and ERK1/2 pathways [22,23,24]. Thus, we determined the effects of CN on the expressions of TNF-α, IL-1β, and IL-6 and the activation of NF-κB and ERK1/2 by HMGB1. Data showed that CN inhibited HMGB1-induced expressions of TNF-α, IL-1β, and IL-6 and the activation of NF-κB and ERK1/2 (Figure 6A–F). Moreover, HMGB1 enhanced the subnuclear localization of p65 NF-κB in HUVECs, which was downregulated by the application of CN (Figure 6G).

### 2.6. Overexpression of HMGB1 Prevents the Anti-Inflammatory Functions of CN

**Figure 7:**
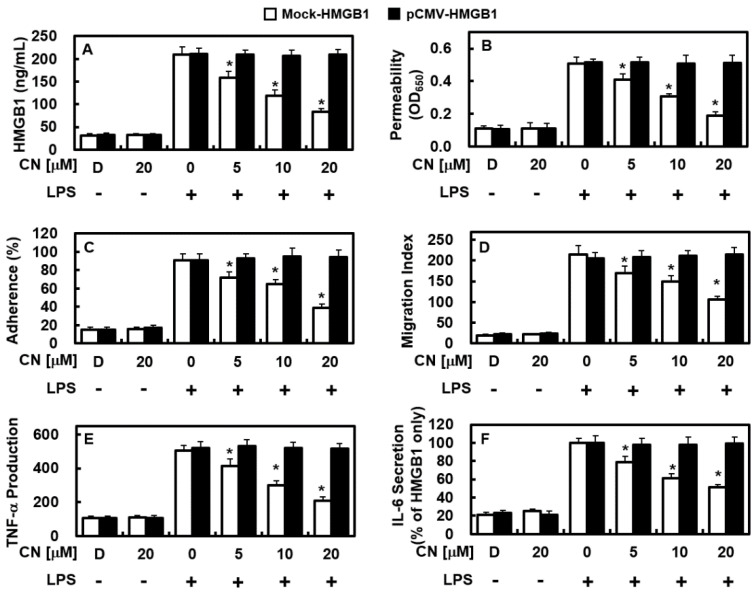
Effects of overexpression of HMGB1 on the anti-inflammatory functions of CN. Overexpression of human HMGB1 in HUVECs was achieved using the pCMV6-Ac-GFP vector (pCMV-HMGB1). (**A**–**F**) represent similar experiments as shown in Figure 2A (**A**), Figure 4A (**B**), Figure 5B (**C**), Figure 5C (**D**), Figure 6A (**E**), and Figure 6B (**F**), except that HUVECs were transfected with mock-vector (Mock-HMGB1) or pCMV6-Ac-GFP vector (pCMV-HMGB1). Data are expressed as the mean ± SD values of three independent experiments on different days. * *p* < 0.05 versus group treatment with LPS only.

Then, we investigated if hyperexpression of HMGB1 could inhibit the ameliorative effect of CN on inflammation. In this procedure, overexpression of the human HMGB1 in endothelial cells was achieved by inserting human HMGB1 into the pCMV6-Ac-GFP vector (pCMV-HMGB1). Overexpression of HMGB1 in endothelial cells was confirmed by using real-time PCR (Supplementary Figure S1). As shown in Figure 7A, HMGB1 overexpression in pCMMV-HMGB1 cells treated with LPS was not suppressed by CN, whereas increased HMGB1 release in Mock-HMGB1 by LPS was suppressed by CN, which appeared the same as Figure 2A. Moreover, the integrity stabilizing (Figure 7B), anti-adhesiveness (Figure 7C), and anti-translocation (Figure 7D) effects of CN on inflammatory immune cells was inhibited in HMGB1 overexpression in LPS-induced pCMMV-HMGB1. In addition, the suppressive effects of CN on the expressions of TNF-α (Figure 7E) and IL-6 (Figure 7F) were ameliorated in HMGB1-overexpressing cells. These results suggest that hyperexpression of HMGB1 and the subsequent release of LPS-induced HMGB1 was not prevented by CN in cells carrying the pCMV6-Ac-GFP vector (Figure 7A). Consequently, CN was unable to manage LPS-induced lethal inflammation activity, such as excessive permeability, adherence, translocation of inflammatory immune cells, and the expression of TNF-α and IL-6 (Figure 7). In addition, aspects like these were still challenging for CN, even at higher doses (Supplementary Figure S2). Although HMGB1-induced inflammatory activity could be prevented or protected when HMGB1 was knocked down/out in endothelial cells, other types of inflammation-related factors may have caused comparable inflammatory activity.

### 2.7. Effects of CN Administration on the Survival Rate and Reduced Tissue Injury in CLP-Induced Septic Mice

**Figure 8:**
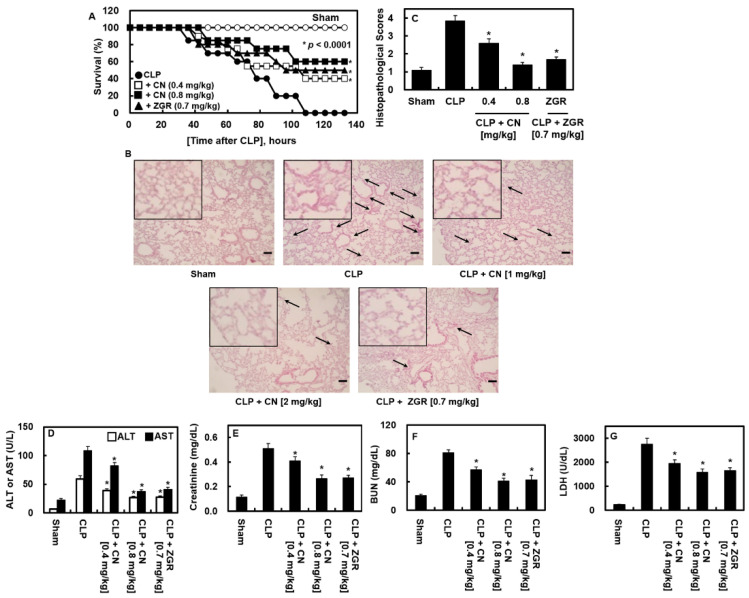
Effects of CN on lethality and tissue injury after the CLP procedure. (**A**) The suppressive effects of CN on mice lethality induced by CLP were monitored every 12 h for 132 h after CLP. Sterile saline was administered to sham-operated mice (○) and control CLP mice (●) (*n* = 20); (**B**) representative photographs of lung tissues in panel C (H&E staining, 200×). CLP surgery and drug administration conditions are as shown in (**A**). Samples were collected after 4 days. The arrows indicate leukocyte infiltration. The black box picture in the figure is a part of the enlarged picture; (**C**) The histological analysis of the effects of CN on lung injury induced by CLP (*n* = 5). The histopathological scores of the injured lung tissues were counted and evaluated as described; (**D**–**G**) The suppressive effects of CN on the plasma levels of various organ injury markers: (**D**) aspartate transaminase (AST) and alanine transaminase (ALT), (**E**) creatinine, (**F**) blood urea nitrogen (BUN), and (**G**) LDH (*n* = 5). Data are expressed as the mean ± SD of three independent experiments performed on different days with similar results. * *p* < 0.05 versus CLP alone.

Next, we administered CN to CLP operated mice to examine the preventive role of CN in the survival of mice with sepsis generated by CLP surgery. As the sole administration of CN or ZGR did not protect against CLP-induced lethality (data not shown), we injected CN (0.4 or 0.8 mg/kg) or ZGR (0.7 mg/kg) at 12 h and 50 h for 132 h after CLP. The mice were examined every 12 h. The mice that received two injections of CN showed considerable improvement in survivals after surgery (*p* < 0.00001, Figure 8A). The results showed that, even if HMGB1 expression was markedly decreased by CN administration once at 12 h after CLP (Figure 2B), two injections were necessary to suppress HMGB1-induced inflammation activity and improve animal survival. Our results indicated that CN may be an effective candidate for the treatment of sepsis-related pathology.

Acute inflammation, which occurs globally during septic deterioration, often leads to MOF and the accompanying injury to critical organs in the pulmonary and renal systems [25]. The therapeutic activity of CN on CLP-mediated pulmonary damage was also examined. CN mitigated the CLP-mediated pathologic abnormality in lung tissue and sever injury to the tissue (Figure 8B,C). The concentrations of ALT and AST in sera (Figure 8D), which is an indication of the damage to the liver, and of BUN and creatinine (Figure 8E,F), which is an indication of damage to the kidney, were greatly increased by CLP surgery. CN not only downregulated the increase in these markers, but also the elevation of lactate dehydrogenase (LDH), which is another important marker of tissue injury (Figure 8G).

## 3. Discussion

The protective effect of CN on the remarkable vascular barrier disruption reaction in septic state was evaluated. Under normal physiological conditions, the vascular endothelium plays an important role in maintaining the integrity of the vascular barrier in response to the extracellular environment. Therefore, disruption of vascular barrier integrity in the septic response is a major and important process as it can lead to abnormal and severe systemic vascular hyperpermeability and coelomic edema that are prominent in septic patients [26]. Therefore, strengthening the recovery capacity of vascular structure integrity after malignant symptoms and managing vascular consistency are promising pharmacologic approaches for sepsis.

Molecular activities for the exocytosis of HMGB1 can be downregulated in two general ways, i.e., passively and actively [6,27]. Passive secretion is caused by necrosis and programmed cell death, whereas active secretion is caused by the post-translational modification (PTM) of nuclear HMGB1 [6,27]. Previous research [16,28] has suggested that the acetylation of lysine residues within a couple of intranuclear protein loci of HMGB1 was crucial in the PTM response in reaction to pathogenic antigens (such as LPS). Our experimental analysis showed that antigen molecules (LPS) induced the secretion of HMGB1 (Figure 2A) and the acetylation of lysine residues (Figure 3B), suggesting that HUVECs secreted HMGB1 by the active method.

The inhibition of HMGB1 secretion (Figure 2A,B), reduction of the quantity of expressed HMGB1 signal recipients, such as TLR2, TLR4, and RAGE (Figure 2C), and the HMGB1-induced excessive permeability (Figure 4A–C) through blocking the p38 biochemical modification (Figure 4D), along with its pathway by CN could influence the progression of HMGB1-induced sepsis. Furthermore, CN suppressed the reaction that occurs between the inflammatory immune cells and endothelial cells by downregulating the adhesion molecules (i.e., E-selectin, VCAM, and ICAM (Figure 5)). By inhibiting HMGB1 release, CN significantly decreased the level of proinflammatory cytokines (i.e., TNF-α, β, IL-6, and IL-1β (Figure 6A–C)), the response of the transcription factors NF-κB and ERK1/2 (Figure 6E,F) in inflammation, and the transfer of NF-κB to the extranuclear matrix (Figure 6G). These HMGB1-mediated modifications and HMGB1 itself, as well as other mediators involved in the inflammatory signaling pathway, were inhibited by the protective action of CN.

In sepsis, the NF-κB response in endothelial cells indicates multisequential pathways that cause hyperpermeability; the production of biochemical substances, such as cytokines, chemokines, and enzymes; and the elevation of CAMs, which are modulated by NF-κB [29,30]. The downregulation of the NF-κB pathway, especially in the endothelium, can protective against inflammation [31]. NF-κB suppression causes a decrease in CAMs and other inflammation-related factors in the vascular barrier, resulting in resistance to the induction of inflammatory immune cells and relieving the symptoms of sepsis [32,33].

More than a decade ago, it was demonstrated that CN has counter-inflammatory effects on the human endothelium [9] and an ameliorative impact on severe inflammatory conditions in immune phagocytes in vitro and in vivo [9], which was confirmed in this research. CN administration inhibited the subcellular localization of NF-κB and decreased not only the expression of CAMS [9], but also TNF-α, IL-6, and nitric oxide generation, as the increased concentration of CN stimulated phagocytes and additionally improved survival rate of CLP animal models. However, our research differs from previous research in several ways: (1) the quantity of treated CN; (2) the inhibitory effect of CN on HMGB1-related responses in sepsis; (3) the restorative effect of CN on the endothelial cell wall; and (4) the interpretation of the biochemical reactions that are affected by the anti-inflammatory effect of CN. The CN dose used by Jiang et al. that presented an ameliorative effect on inflammation was between 10^−9^ M and 10^−7^ M in the in vitro experiments and 25 or 50 mg/kg in the in vivo experiments [34]. These are different effective concentrations; indeed, the concentrations applied in the in vitro assay were more dilute than those used in our research, but they were more concentrated than those used in our in vivo assay. Notably, CN doses from 10^−9^ M to 10^−7^ M for in vitro analysis are more dilute than in other studies [9,10,35,36].

The main limitation of this study was the inability to determine the pharmacokinetics of CN in vivo. After CN is injected intravenously, it undergoes absorption, distribution, metabolism, and excretion (ADME), which describes the disposition of a pharmaceutical compound within an organism. These four criteria all influence the levels, kinetics, and overall performance of the drug. Thus, additional research is required to elucidate the pharmacokinetic properties of CN in vivo.

CN results in the inhibition of HMGB1-induced vascular structure disruption by reducing HMGB1 secretion in LPS-stimulated endothelial cells, downregulating the CLP-induced secretion of HMGB1, and improving vascular wall stability. Furthermore, the restorative effect of CN on the vascular wall structure was shown by CN treatment of CLP-induced mice. In this assay, the administration of CN improved the survival rate of CLP-induced mice and ameliorated tissue/organ injuries. Our results suggest that CN may be a promising candidate for the clinical treatment of septic diseases and severe systemic inflammation.

## 4. Materials and Methods

### 4.1. Cell Culture and Reagents

Primary HUVECs were produced by Cambrex Bio Science (Charles City, IA, USA) and managed using previously established methods [37,38]. Cells at passage 3–5 were used for all experiments. We purchased CN (Figure 1, purity > 96%), ZGR (purity > 96%), LPS (serotype 0111:B4, L5293, from *Escherichia coli*), crystal violet, Evans blue dye, MTT, penicillin G and streptomycin, mercaptoethanol, and DMSO from Sigma Chemical Co. (St. Louis, MO, USA). Genetically processed human HMGB1 was obtained from Abnova (Taipei City, Taiwan). For the vehicle control, and for dissolving CN or ZGR, 0.5% dimethyl sulfoxide (DMSO) was used. CN was used at different doses (1, 2, 5, 10, or 20 μM) for the in vitro assay and for the in vivo assay (0.08, 0.2, 0.4, or 0.8 mg/kg).

### 4.2. Animals and the Cecal Ligation and Puncture (CLP) Procedure

Male C57BL/6 mice (6–7 weeks of age, 27 g) were obtained from Orient Bio Co. (Sungnam, Korea). We prepared the CLP mouse model as determined in a former protocol [23]; the sham group consisted of exposure of the cecum but no ligation or puncture. The prepared samples were injected intravenously at a dose of 0.08, 0.2, 0.4, or 0.8 mg/kg for CN or 0.7 mg/kg ZGR at 24 h after CLP surgery. The release of HMGB1, cell integrity assay, and immune cell migration was examined. To estimate survival rate, a sample of 0.4 or 0.8 mg/kg CN or 0.7 mg/kg ZGR was injected intravenously at 12 and 50 h after CLP (Figure 8). These animal experiments were permitted by the Animal Care Committee at Kyungpook National University before research was started (IRB no. KNU 2020-107).

### 4.3. Competitive Enzyme-Linked Immunosorbent Assay for HMGB1

The release of HMGB1 into mouse serum or the culture fluid of cell was examined by a competitive enzyme-linked immunosorbent assay (ELISA), as described previously [39,40].

### 4.4. Expression Levels of Cell Adhesion Molecules (CAMs) and HMGB1 Receptors

The expression levels of vascular cell adhesion molecule (VCAM)-1, intercellular CAM (ICAM)-1, and E-selectin were assessed using whole-cell ELISAs, as described elsewhere [12]. A single layer of HUVECs of 80–90% confluence was stimulated with HMGB1 (1 μg/mL) for 16 h (to assess the level of VCAM-1 and ICAM-1) or 22 h (to assess the level of E-selectin). Then, CN or ZGR treatment was applied. Specific antibodies (A-9, H-80, and A-9, Santa Cruz Biotechnology Inc., Dallas, TX, USA) were used for assaying the expression levels of Toll-like receptor (TLR)2, TLR4, and the receptor for advanced glycation end products (RAGE).

### 4.5. Cell Viability Assay

The cultured HUVECs were incubated with LPS (100 ng/mL) for 16 h or CN for 48 h and the 3-(4,5-dimethylthiazol-2-yl)-2,5-diphenyltetrazolium bromide (MTT) assay was performed to estimate cell viability [40,41,42].

### 4.6. Preparation of Cytoplasmic and Nuclear Extracts and Western Blotting Analyses

The processes of centrifugation, immunoprecipitation, and Western blotting were performed to acquire cytoplasmic and nuclear derivatives from the cell pellets stored on ice, as described previously [39]. The antibodies for actin and lamin B were used as the loading controls for Western blotting analysis of the cytoplasmic and nuclear fractions.

### 4.7. Cell-Cell Adhesion Assay

The following description covers the process used to estimate the adhesion between human neutrophilic leukocytes and HUVECs [12]. First, each layer of HUVECs was stimulated with HMGB1 (1 μg/mL) for 16 h, and then incubated with 20 µM ZGR or escalating concentrations of CN for 6 h. Neutrophilic leukocytes (3 × 10^5^ cells/well) were stained with Vybrant DiD dye, and then coated with a layer of activated HUVECs. The comparative quantity of stained coated cells was estimated spectrophotometrically from the fluorescence emission (total signal) of Vybrant DiD dye using a microplate reader (Tecan, GmbH, Grödig, Austria). Following the incubation of neutrophilic leukocytes and HUVECs for 1 h, non-adhesive cells were removed by rinsing the cells gently with PBS four times. Then, the fluorescence emission from the remaining cells was measured again (i.e., the adherent signal). The proportion of neutrophilic leukocyte adhesion to HUVECs was evaluated using the following formula: % adherence = (adherent signal/total signal) × 100.

### 4.8. Permeability Assay

To examine the impact of CN and ZGR on cell permeability in vitro, endothelial permeability was examined using a bi-compartmental chamber, as described previously [12]. For the in vivo investigation, male mice were administered HMGB1 (2 μg/mouse) by intravenous injection. After 16 h, CN compound (0.08, 0.2, 0.4, or 0.8 mg/kg) or ZGR (0.7 mg/kg) was subsequently injected. The amount of intravenously injected Evans blue dye leaked into the abdominal cavity per mouse and standard curve of this dye were used to estimating the precise flux through vascular structure, as described elsewhere [12].

### 4.9. In Vitro Migration Assay

The level of human neutrophilic leukocytes migration through HUVECs was measured in accordance with an existing report [12]. The assays were conducted in 6.5 mm transwell plates, which consist of a filtering membrane with a pore size of 8 µm. To acquire a suitably confluent monolayer of endothelial cells, HUVECs were incubated for 72 h. Before the neutrophilic leukocytes were applied to the upper chamber, HUVECs were stimulated with HMGB1 (1 μg/mL) for 16 h and incubated with 20 µM ZGR or increasing concentrations of CN for 6 h. Later, the transwell plates were incubated at 37 °C for 2 h and the neutrophilic leukocytes that were not attached and did not migrate, and HUVECs remaining in the upper compartment and on the filter were removed. A solution of 8% glutaraldehyde was used to fix the cells and 0.25% crystal violet in 20% methanol (*w*/*v*) was used to stain the neutrophilic leukocytes translocated through the filter membrane. Cells in nine randomly selected high-power microscopic fields (200×) were counted. All assays were repeated twice per well on duplicate wells of the same test conditions and the outcomes were reported as the degree of migration. Nine randomly selected high power microscopic fields (HPF, 200×) were counted and expressed as a migration index.

### 4.10. In Vivo Leukocyte Migration Assay

We used 2% isoflurane (Forane, JW Pharmaceutical, Seoul, Korea) in oxygen which was administered through an inhaling chamber first, and through a facemask and veterinary anesthesia equipment for small rodents (RC2, Vetequip, Pleasanton, CA, USA) to tranquilize the mice. The in vivo translocation of leukocytes, the immunocytes, was analyzed as described elsewhere [11,43]. The mice were first administered with HMGB1 (2 μg/mouse, i.v.) and then, 16 h later, injected with CN (0.08, 0.2, 0.4, or 0.8 mg/kg) or ZGR (0.7 mg/kg). Subsequently, the mice were euthanized by dislocation of the cervical spine, and the abdominal cavity was rinsed with PBS (5 mL). The acquired abdominal liquid substances (20 μL) were dyed with 0.38 mL of Turk’s solution (0.01% crystal violet in 3% acetic acid) and the number of cells was counted using an optical microscope.

### 4.11. ELISAs for Phosphorylation of p38 MAPK/SAPK, NF-κB, TNF-α, ERK 1/2, IL-1β, and IL-6

The activation of p38 mitogen-activated protein kinase/stress-activated protein kinase (MAPK/SAPK) is determined by the quantity of p38 MAPK/SAPK that is phosphorylated in the pathway. The level of phosphorylation was estimated using an ELISA kit (Cell Signaling Technology, Danvers, MA, USA). In addition, the expression of IL-1β, IL-6, and TNF-α in the cell culture supernatants and the total and phosphorylated levels of the extracellular signal-regulated kinase (ERK) 1/2 (R&D Systems, Minneapolis, MN, USA) and p65 subunit of nuclear factor kappa B (NF-κB) (Cell Signaling Technology, Danvers, MA, USA) in the nuclear lysates were detected using appropriate ELISA kits.

### 4.12. Transfection for Stable Human HMGB1-Overexpressed HUVECs

Overexpression of HMGB1 in HUVECs was achieved by incorporating human HMGB1 in the pCMV6-Ac-GFP vector (RG205918, OriGene Technologies, Inc., Rockville, MD, USA). To insert DNA safely and stably, HUVECs were plated in a 12-well plate at 50–60% confluency, cultivated until the next day with 5 µg plasmid DNA using Lipofectamine 3000 (Invitrogen, USA), in accordance with the manufacturer’s protocols. After recombination, the cells were cultivated in culture fluid containing neomycin (4 mg/mL). The cells with neomycin resistance were observed 3 weeks after cell recombination.

### 4.13. H&E Staining and Histopathological Examinations

Male mice subjected to CLP surgery were used for the animal model of sepsis and were administered an intravenous injection of CN (0.4 or 0.8 mg/kg) or ZGR (0.7 mg/kg) at 12 and 50 h after the surgery. All mice were euthanized after 4 days. Blinded analysis with an optical microscope was conducted to observe the histologic state of the lung tissue, by examining vascular structure, the migration of immune cells to represent inflammation, and tissue edema, as described previously [11]. The results were classified into four grades: Grade 1 indicated normal histopathology; Grade 2 indicated minimal neutrophil leukocyte infiltration; Grade 3 indicated moderate neutrophil leukocyte infiltration, perivascular edema formation, and partial destruction of pulmonary architecture; and Grade 4 indicated dense neutrophil leukocyte infiltration, abscess formation, and the complete destruction of pulmonary architecture.

### 4.14. Measurement of Tissue Injury Markers

Albumin depleted plasma was accomplished with a commercially available kit (Albumin Removal Kit, Thermo Fisher Scientific, Waltham, MA, USA). The plasma concentrations of blood urea nitrogen (BUN), creatinine, alanine transaminase (ALT), aspartate transaminase (AST), and lactate dehydrogenase (LDH) were detected using commercially available products (Pointe Scientific, Lincoln Park, MI, USA).

### 4.15. Statistical Analysis

In general, data were presented as the mean ± standard deviation (SD) derived from three respective trials, unless otherwise stated. To compare the data between several groups, ANOVA and Tukey’s post hoc tests were applied for statistical analysis. *p* values of <0.05 were considered to be statistically significant. The Kaplan–Meier curve was used to distinguish the difference in survival rate in the CLP model mice and control mice.

## Data Availability

The data presented in this study are available upon reasonable request from the corresponding author.

## Supplementary Materials

The following are available online at https://www.mdpi.com/article/10.3390/ijms23042065/s1.
