# Supplementary material for: Anti-Septic Functions of Cornuside against HMGB1-Mediated Severe Inflammatory Responses
Source: Int J Mol Sci. 2022 Feb 13;23(4):2065. doi: 10.3390/ijms23042065 (PMC8874448; doi:10.3390/ijms23042065)
Supplement: Supplementary file 1 [file ijms-23-02065-s001.zip › ijms-1579476-supplementary.pdf]

The effects of CN on HMGB1 release by LPS (100 ng/mL, 16 h) action in HUVECs .

*p < 0.05 versus group treated with LPS alone .

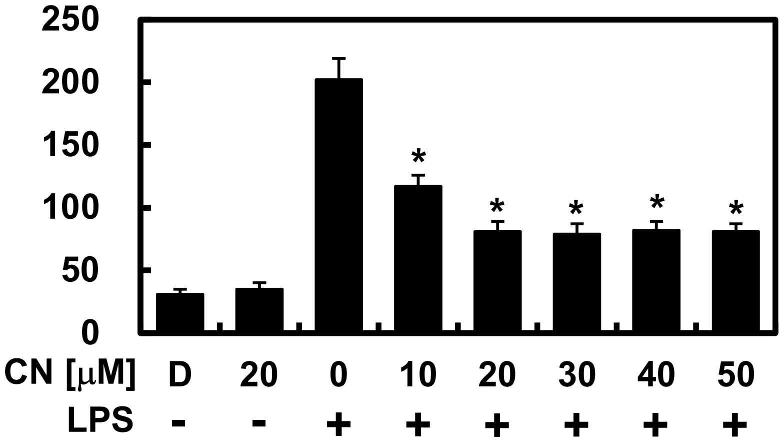

Supple Figure S1

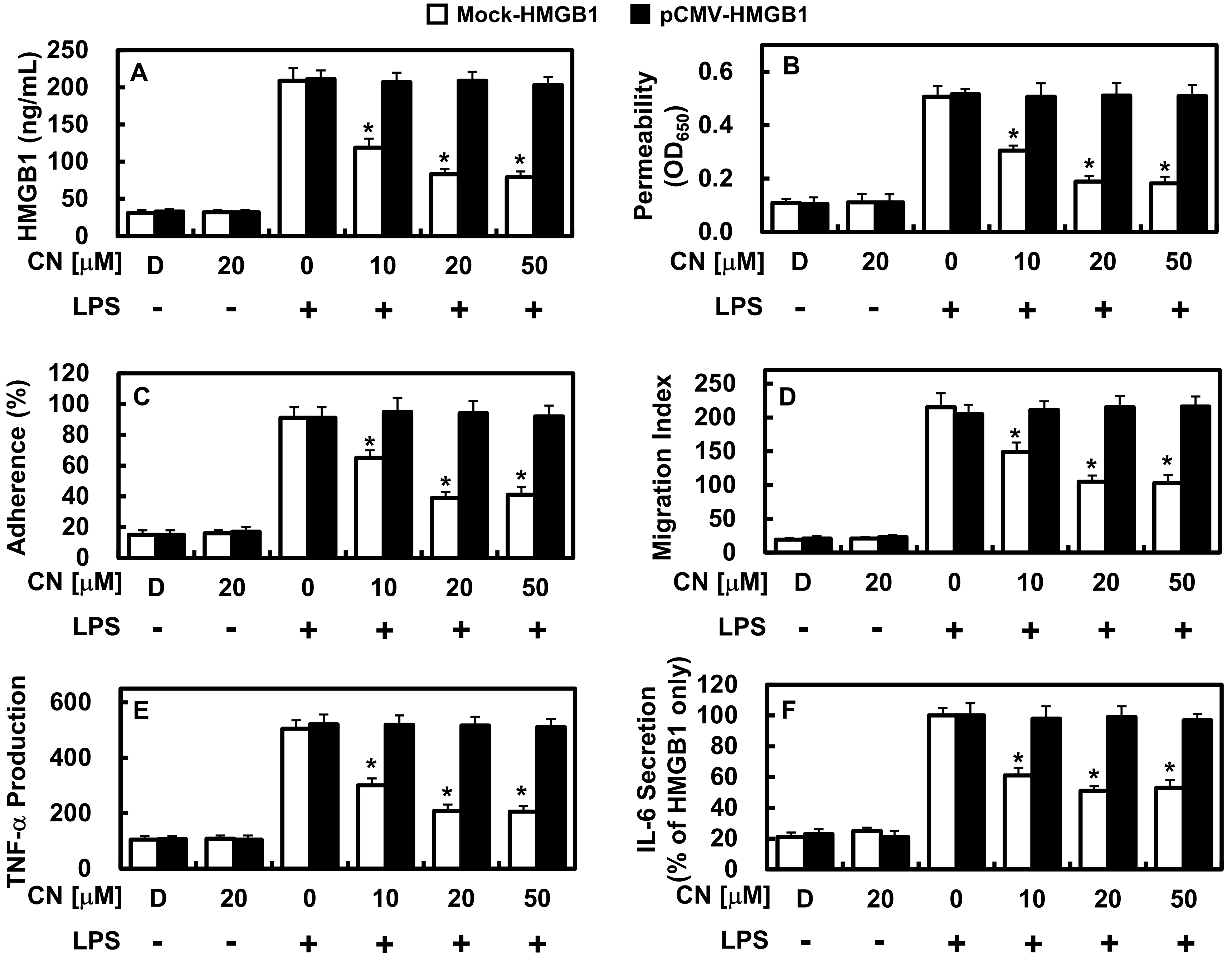

Supple Figure S2
